# Biochemical and molecular heterogeneity among isolates of *Yersinia ruckeri* from rainbow trout (*Oncorhynchus mykiss*, Walbaum) in north west Germany

**DOI:** 10.1186/1746-6148-9-215

**Published:** 2013-10-21

**Authors:** Yidan Huang, Martin Runge, Geovana Brenner Michael, Stefan Schwarz, Arne Jung, Dieter Steinhagen

**Affiliations:** 1 Fish Disease Research Unit, University of Veterinary Medicine Hannover, Foundation, Hannover, Germany; 2 Lower Saxony State Office for Consumer Protection and Food Safety (LAVES), Food and Veterinary Institute Braunschweig/Hannover, Hannover, Germany; 3 Institute of Farm Animal Genetics, Friedrich-Loeffler-Institute (FLI), Neustadt-Mariensee, Germany; 4 Clinic for Poultry, University of Veterinary Medicine Hannover, Foundation, Hannover, Germany

**Keywords:** Enteric Red mouth Disease, REP-PCR, ERIC-PCR, BOX-PCR, PFGE, Non-motile strains

## Abstract

**Background:**

Enteric Redmouth Disease (ERM), caused by *Yersinia ruckeri*, is one of the most important infectious diseases in rainbow trout (*Oncorhynchus mykiss*) aquaculture in Europe. More recently, non-motile vaccine resistant isolates appear to have evolved and are causing disease problems throughout Europe, including Germany. The aim of this study was to analyse the variation of biochemical and molecular characteristics of *Y. ruckeri* isolates collected in north west Germany as a basis for strain differentiation. The isolates originated mainly from rainbow trout and were characterised by biochemical profiling, 16S rDNA sequencing, repetitive sequence-based PCRs, including (GTG)_5_-PCR, BOX-PCR, ERIC-PCR and REP-PCR, and pulsed-field gel electrophoresis (PFGE).

**Results:**

In total, 83 isolates were characterised, including 48 isolates collected during a field study in north west Germany. All isolates were confirmed as *Y. ruckeri* by the API 20E system. Five isolates were additionally confirmed as *Y. ruckeri* by *Y. ruckeri*-specific PCR and 16S rDNA sequencing. Only 17 isolates hydrolyzed Tween 80/20. Sixty-six isolates (79.5%) were non-motile. Two different patterns were obtained by REP-PCR, five patterns by ERIC-PCR, four patterns by (GTG)_5_-PCR and three patterns by BOX-PCR. *NotI*-directed PFGE resulted in 17 patterns that differed from each other by 25–29 fragments. Isolates from the field study clustered together as PFGE type C. According to the results of API 20E, repetitive sequence-based PCRs and PFGE, these isolates could be subdivided into 27 different groups.

**Conclusions:**

The detailed molecular and phenotypic characterisation scheme developed in this study could be used to help trace the dissemination of *Y. ruckeri* isolates, and thus may represent part of improved disease monitoring plans in the future.

## Background

Enteric Red mouth Disease (ERM), caused by *Yersinia ruckeri*, is one of the most important infectious diseases in rainbow trout (*Oncorhynchus mykiss*) aquaculture in Europe. Signs of a clinical infection of rainbow trout may include haemorrhages in various tissues and organs, particularly around the mouth, in the gills, muscles, peritoneum and the lower intestine. The bacterium has been isolated from various fish species, including rainbow trout, steelhead (*Oncorhynchus mykiss*), lake trout (*Salvelinus namaycush)*, cutthroat (*O. clarkii)*, brown (*Salmo trutta)* and brook trout (*S. fontinalis)*, coho (*O. kisutch)*, sockeye (*O. nerka)*, Chinook (*O. tshawytscha)* and Atlantic salmon (*Salmo salar*) [1]. In Europe, it is endemic in many trout farms and can cause high economic losses [2-6]. The disease most commonly affects younger rainbow trout at temperatures above 10°C. Outbreaks are often related to adverse situations or stressed carrier fish which shed the pathogen and facilitate the onset of an infection. In infected trout populations, ERM can be controlled effectively by antibiotic treatment and by the application of inactivated whole cell vaccines [7], which are available commercially and are considered to be successful [8]. Nevertheless, outbreaks of ERM are still periodically observed, especially in endemic areas.

Among *Y. ruckeri* isolates, several serological variations have been reported [9-11]. While initially most outbreaks of ERM were caused by *Y. ruckeri* isolates of serotype O1 (Hagerman strain), *Y. ruckeri* isolates of different serotypes have been more recently obtained from sick fish during outbreaks of ERM in the UK, Spain and the U.S.A. [12-14]. Moreover, several of the commercial ERM vaccines seemed to not provide sufficient protection against infections caused by some of these isolates [13,14]. All these ‘vaccine-tolerant’ isolates lacked motility (assigned as biotype 2, BT2 isolates), while isolates from previous outbreaks were all motile (biotype 1) [13]. It was hypothesised that vaccination resulted in a selective pressure that enabled the emergence of non-motile isolates that are tolerant to the commercial vaccines. Therefore, there is a high risk that these non-motile vaccine-tolerant isolates spread and cause severe outbreaks of disease in trout farms. Wheeler *et al.*[15] found that the BT2 isolates in mainland Europe have arisen independently from the local BT1 isolates. Recently, Welch *et al.*[16] indicated that the European BT2 isolates were not from the same clonal group. In Germany, the earliest report about BT2 isolates was published in 1994 [17]. Recently, ‘vaccine-tolerant’ *Y. ruckeri* were isolated from outbreaks in trout hatcheries in north west Germany, in particular in the federal state of North Rhine-Westphalia (NRW).

In order to avoid further spreading of these ‘vaccine-tolerant’ isolates, an epidemiological survey on the occurrence and further phenotypic and molecular characterisation of *Y. ruckeri* from trout hatcheries was performed. In this study, interspecies differences of *Y. ruckeri* isolates from north west Germany were detected via biochemical and molecular traits to determine how related isolates from different origins are. For this, several typing methods were compared for their ability to differentiate *Y. ruckeri* isolates from rainbow trout and other fish species.

## Results

### Biochemical and physiological characterisation

Among the variable biochemical characteristics of all 83 *Y. ruckeri* isolates (Table 1), gelatine hydrolysis, Voges-Proskauer (VP) reaction and Tween hydrolysis were the most variable biochemical reactions. The results of VP, nitrate production, citrate utilization and gelatine hydrolysis from both, conventional biochemical analysis and testing by the API 20E system, were listed also in Table 1. For motility, Tween 80/20 hydrolysis and citrate utilization, the isolates obtained in Lower Saxony (LS) from fish species other than rainbow trout were more similar to the reference strain DSM 18,506 than the isolates obtained from rainbow trout hatcheries in NRW and LS. Only 16 isolates hydrolysed Tween 80/20. For these isolates motility was confirmed by microscopical inspection as well as by cultivation in API M medium. Those isolates were recognized as non-motile isolates. In particular, isolates collected from trout hatcheries in NRW were lacking flagella and motility; 92% of the isolates from this federal state were non-motile (Table 1).

**Table 1:** **Biochemical characteristics of *Yersinia ruckeri *** **isolates from fish in aquaculture in north west Germany**

**Test**	**Reference strain (DSM 18,506)**	**Number of the field isolates tested positive (%)**				
		**Total**	**Isolates from rainbow trouts in H**	**Isolates from rainbow trouts in N**	**Isolates from rainbow trouts in L**	**Isolates from other fish in L**
		**(n = 82)**	**(n = 8)**	**(n = 49)**	**(n = 19)**	**(n = 6)**
Motility	+	16 (19.5)	1 (12.5)	4 (8.2)	6 (31.6)	5 (83.3)
Nitrate reduction	+	77 (93.9)	8 (100.0)	47 (95.9)	17 (89.5)	5 (83.3)
Nitrate reduction *	-	72 (87.8)	8 (100)	45 (91.8)	14 (73.7)	5 (83.3)
Citrate utilization	+	59 (72.0)	6 (75.0)	32 (65.3)	15 (78.9)	6 (100)
Citrate utilization *	+	50 (61.7)	6 (75.0)	29 (59.2)	10 (52.6)	5 (83.3)
Voges-Proskauer	-	48 (58.5)	5 (62.5)	29 (59.2)	10 (52.6)	4 (66.7)
Voges-Proskauer*	-	52 (63.4)	4 (50.0)	37 (75.5)	7 (36.8)	4 (66.7)
Gelatine hydrolysis	-	81 (98.8)	8 (100.0)	49 (100.0)	19 (100.0)	5 (83.3)
Gelatine hydrolysis*	-	77 (93.9)	8 (100.0)	48 (98.0)	16 (84.2)	5 (83.3)
Methyl-red	+	76 (92.7)	7 (87.5)	48 (98.0)	18 (94.7)	3 (50.0)
Tween 20 hydrolysis	+	16 (19.5)	1 (12.5)	4 (8.2)	6 (31.6)	5 (83.3)
Tween 80 hydrolysis	+	16 (19.5)	1 (12.5)	4 (8.2)	6 (31.6)	5 (83.3)
Acid from sorbitol	-	5 (6.1)	1 (12.5)	1 (2.0)	0 (0.0)	3 (50.0)

* API 20E test system.

L: Lower Saxony, H: Hessen, N: North Rhine-Westphalia.

In the API 20E test, 27 isolates showed the numeric profile 5,107,100, 23 isolates with the numeric profile 5,306,100. These profiles differed from that of the reference strain DSM 18,506 with the profile 5,304,100. For a biochemical typing of the isolates, typing numbers from “a” to “g” were assigned to individual isolates according to their profile number (Table 2).

**Table 2:** **Different genetic groups of *Y. ruckeri***

**Patterns from different genotypic method**^***a***^					**API 20E profile**^***b***^	**No. of isolates**	**Typing Group (tp)**
**REP-PCR**	**BOX-PCR**	**(GTG)**_**5**_**-PCR**	**ERIC-PCR**	**PFGE**			
R1	B1	G1	E1	Pt C1	a	5	tp1
					b	21	tp2
					c	1	tp3
					d	1	tp4
					h	2	tp5
				Pt C10	a	8	tp6
					b	1	tp7
					c	7	tp8
					d	1	tp9
					g	1	tp10
				Pt C4	c	1	tp11
				Pt C6	g	1	tp12
				Pt C8	b	2	tp13
				Pt C9	a	1	tp14
				Pt C3	b	1	tp15
				Pt C7	c	3	tp16
				Pt C5	d	1	tp17
				Pt C2	a	7	tp18
					b	2	tp19
					c	7	tp20
				Pt C11	a	2	tp21
				Pt C12	c	1	tp22
		G 2	E 3	Pt A1	e	1	tp23
	B 2	G4	E 4	Pt A2	e	1	tp24
	B 3	G3	E 2	Pt B1	f	1	tp25
R 2	B 1	G1	E 5	Pt B2	e	2	tp26
R 3	B 1	G 1	E 6	Pt D1	g	1 (DSM18506)^*b*^	tp27

^*a*^PCR-based typing methods: REP-PCR, repetitive extragenic palindromic-PCR; BOX-PCR, BOX-A1R-based repetitive extragenic palindromic-PCR; (GTG)_5_-PCR, polytrinucleotide (GTG)_5_-PCR; ERIC-PCR, enterobacterial repetitive intergenic consensus-PCR. Macrorestriction-based typing method, PFGE macrorestriction analyis by pulsed-field gel electrophoresis.

^*b*^Reference strain DSM 18506.

^*c*^API 20E profiles were as follows: a (5,306,100, n = 23), b (5,107,100, n = 27), c (5,307,100, n = 20), d (5,106,100, n = 3), e (5,307,500, n = 4), f (5,305,700, n = 1), g (5,304,100, n = 2 + reference strain DSM 18,506), h (5,104,100, n = 2).

### Genetic characterisation

**Figure 1:**
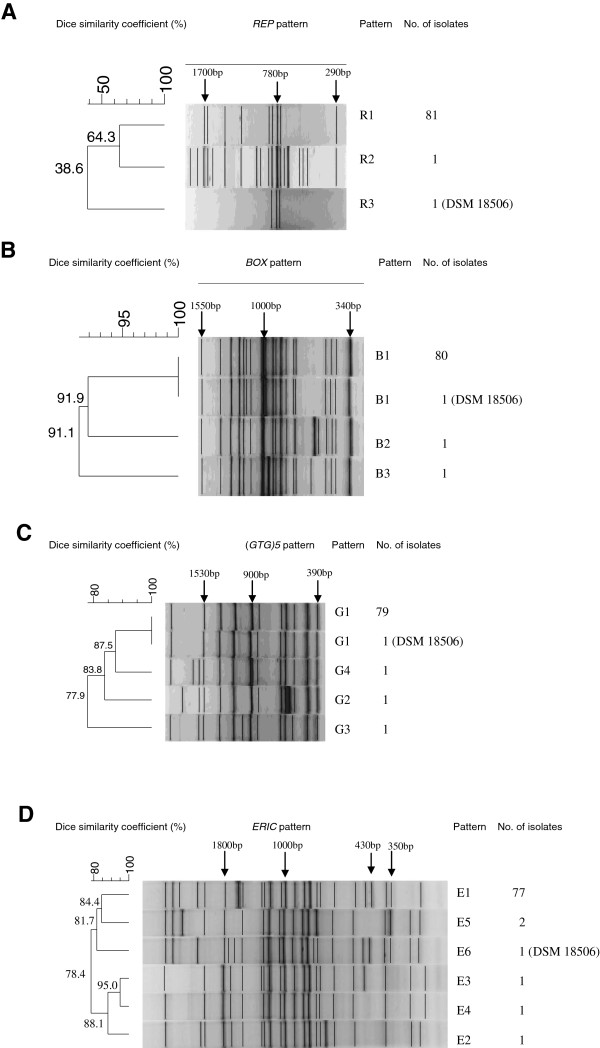
**A dendrogram of *Y. ruckeri *** **isolates constructed using the UPGMA method (tolerance 1%) using Gel Compar II (Applied Maths), based on fingerprints of different repetitive sequence-based PCRs: A, REP-PCR; B, BOX-PCR; C, (GTG)5-PCR; D, ERIC-PCR.**

When the isolates were analysed by repetitive sequence-based PCR assays, two amplicon patterns were obtained after REP-PCR, five patterns by ERIC-PCR, four patterns by (GTG)_5_-PCR and three patterns by BOX-PCR (Figure 1). The similarity rates in REP-PCR were clearly lower than in the other repetitive sequence-based PCRs. In BOX-PCR and (GTG)_5_-PCR, the reference strain DSM 18,506 exhibited the same amplicon patterns as most of the isolates (Figure 1). By REP-PCR, 3 or 19 bands were seen in the size range of 200–2000 bp; by BOX-PCR, 18–19 bands were found distributed between 340–1550 bp; by (GTG)_5_-PCR, 11–13 bands were detected from 390–2500 bp; and by ERIC-PCR, 20–25 bands were present in the range from 200–2000 bp (Figure 1). Five isolates, which were positive in sorbitol fermentation, did not exhibit the 430 bp amplicon in ERIC-PCR. Only five isolates from fish species other than rainbow trout showed some differences in repetitive PCRs. These five isolates were additionally confirmed as *Y. ruckeri* (identities = 100%, data not shown) by 16S rDNA sequencing; while the nucleotide sequence of the reference strain DSM 18,506 was not completely identical to the sequence reported from the strain ATCC 29,473 (identities = 99%, data not shown). Conversely, all the isolates from rainbow trout showed rather uniform patterns of repetitive sequence-based PCR.

**Figure 2:**
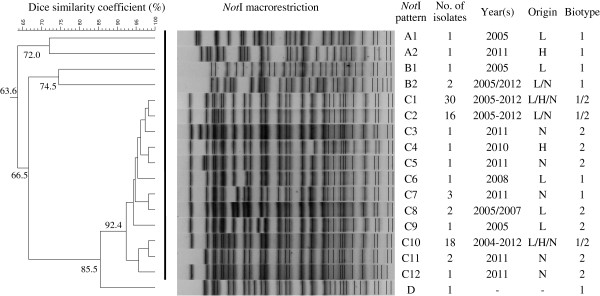
**Dendrogram of *Y. ruckeri *isolates based on *Not*I-macrorestriction (PFGE) patterns.** The similarity analysis was performed using the Dice coefficient and UPGMA method (tolerance 1%). Pulsotype D: reference strain DSM18,506. The similarity of the isolates obtained from Lower Saxony (L), Hessen (H) and North Rhine-Westphalia (N) are given as percentages at the major junctions in the dendrogram. The *Not*I-patterns, number of isolates, year(s) of isolation, geographical origin and biotype (biotype 1: motile, biotype 2: non-motile) are shown.

*NotI*-directed PFGE yielded four major PFGE groups A-D. A total of 17 different PFGE types (A1, A2, B1, B2, C1-C12, D) were detected among the 83 *Y. ruckeri* isolates examined (Figure 2, Table 2). Cluster analysis of these PFGE types showed that the isolates from north west Germany were highly uniform, whereby 30 isolates (36.1%) belonged to PFGE type C1 and 18 isolates to PFGE type C10 (Figure 2). Compared with the reference strain DSM 18,506, all the isolates were different. The isolates from rainbow trout were clustered together (PFGE group C). Isolates from pike, koi and brown trout (PFGE groups A and B) showed the greatest variation when compared to rainbow trout isolates. All isolates previously assigned to biotype 2 clustered in PFGE group C. Isolates from this PFGE group were found in the federal states of LS, Hessen (H) and NRW (Figure 2). Most of the isolates were distributed in all three federal states (PFGE type C1 and C10), while isolates of PFGE type C2 were found only in LS and NRW (Figure 2).

### Discriminatory indices of the typing methods applied

As a discriminatory index, Simpson’s index of diversity was calculated for each typing method used to differentiate all *Y. ruckeri* isolates. Discriminatory indices varied distinctly between the different typing methods. REP-PCR had the lowest D value of 0.048. This means that two randomly selected isolates of this test population had a probability of 4.8% of showing a different REP-PCR pattern. Slightly higher *D* values were calculated for BOX-PCR (*D* = 0.071), (GTG)5-PCR (*D* = 0.095) and ERIC-PCR (*D* = 0.140). Highest D values were seen for biotyping via API 20E (*D* = 0.763) and *NotI*-directed PFGE (*D* = 0790). According to their biochemical and molecular characteristics as obtained by testing with API 20E, PFGE and various PCR-based methods, the *Y. ruckeri* isolates from north west Germany could be subdivided into 27 different groups (Table 2). Most of the isolates from north west Germany (30, 36.6%) belonged to the group (R1/B1/G1/E1/PFGE type C1). Additional information about the origins and isolated years were shown in Additional file 1: Table S1.

## Discussion

Since the first isolation of *Y. ruckeri* in Idaho, USA in 1950s, outbreaks of ERM were frequently reported [7,18,19], especially from the aquaculture industry in countries such as Croatia [3], France [4,20], Finland [2], Germany [5], Italy [21], Switzerland [6] and the UK [14,22]. The bacterium was isolated from different host species [2,23,24], including several salmonid species, channel catfish, goldfish, common carp, European eel, coalfish, and perch. Typing of *Y. ruckeri* isolates from various geographic locations or different fish species has been performed for taxonomic or epidemiological purposes and initially it was based on phenotypical characteristics including the results of biochemical and serological tests [10,25,26]. In addition, molecular techniques, including random amplification of polymorphic DNA (RAPD), multilocus sequence typing (MLST) or pulsed-field gel electrophoresis (PFGE) have been applied to study the genetic diversity of *Y. ruckeri* isolates [15,27-29]. In a study on isolates from various geographic origins, including Chile, Peru, U.S.A., UK, and mainland Europe, a considerable intraspecies diversity of *Y. ruckeri* was found on the basis of the phenotypical characteristics obtained from API 20E, lipopolysaccharide (LPS) and outer membrane profiles [30] as well as by genetic analysis using MLST profiles [27].

The isolates from north west Germany investigated in the present study, showed a strong homogeneity of biochemical results and API 20E profiles with some variability in gelatine hydrolysis, VP reaction and Tween hydrolysis. Variation in some biochemical tests for *Y. ruckeri* has been reported in previous studies [27,30,31], but this variation could not be used as a distinguishing characteristic for phenotypical traits such as serotype or biotype [25,30,32]. While the majority of *Y. ruckeri* isolates from farmed rainbow trout in Peru [33] or Atlantic salmon in Chile [34] were motile, only 16 out of 82 isolates from the present study belonged to biotype 1 (motile and lipase positive). All other isolates could not hydrolyse Tween 80/20, were lipase-negative and lacked flagella.

The API 20E profiles 5,306,100, 5,107,100, 5,104,100 were previously obtained from *Y. ruckeri* isolates from south-west Germany [35], the profiles 5,307,100 and 5,304,100 were already reported from Spanish isolates [13]. While the profiles 5,106,100 and 5,307,500 were also previously reported from *Y. ruckeri* isolates, [36] there was no record about an isolate with the numeric profile 5,305,700. However, 16S rRNA sequencing confirmed this isolate as *Y. ruckeri* (identity = 100%, data not shown).

In addition to the biochemical characteristics, some genetic variability was observed in isolates using several repetitive sequence-based PCR techniques including BOX-PCR, ERIC-PCR, (GTG)_5_-PCR, REP-PCR as well as pulsed-field gel electrophoresis. An analysis of the isolates from north west Germany by repetitive sequence based PCRs gave 3 to 6 groups, with the majority of isolates in one group. Similar observations were already reported from Latin American isolates [33,34]. ERIC-PCR showed the highest discriminatory power. While in previous studies both ERIC-PCR and REP-PCR were considered of some value as tools to study ERM epidemiology [34], in our study, REP-PCR showed the lowest discriminatory power and could not differentiate the isolates very clearly. This could depend on the different origin of the isolates: our isolates originated mainly from rainbow trout, while Bastardo *et al.*[34] studied isolates from Atlantic salmon. When *Y. ruckeri* isolates from Peruvian rainbow trout were analysed by REP-PCR, more homogeneous amplicon patterns were observed as well [33].

PFGE has been frequently applied for detailed molecular typing of different bacteria [37-39], as well as for a differentiation among isolates of *Y. ruckeri*[15]. In the present study, the typing results by PFGE showed strong homogeneity among the isolates obtained from farmed fish species in north west Germany. More than half of the *Y. ruckeri* isolates belonged to the two PFGE types C1 and C10, with 36.1% of the isolates sharing the same PFGE type C1. These two PFGE types belong to the group C PFGE cluster, which comprises isolates from rainbow trout and represents the majority of the isolates. The isolates showing PFGE types A1/A2 or B1/B2 also differed from the other isolates in at least some of their repetitive sequences-based PCR patterns (Table 2). In the present study, no distinct differences were observed in PFGE types from biotype 1 and biotype 2 isolates from rainbow trout.

The data presented in this study may suggest that the isolates from north west Germany originated from the same ancestor and could indicate a clonal population structure. In a previous study on the genetic diversity of *Y. ruckeri* isolates, analysed by MLST, a large number of isolates also belonged to just two sequence types (ST), which formed one clonal complex with 21 out of 30 recognised STs [27]. However, the MLST analysis revealed that genetic recombination appeared to play a greater role than mutation for the generation and maintenance of genetic diversity within the population of *Y. ruckeri*[27]. This would be contradictory to the concept of clonal expansion of a population from a common ancestor that diversified mainly by mutation rather than recombination [40]. MLST results from *Y. ruckeri* rather suggest an epidemic population structure, as it was observed for instance for *Vibrio parahaemolyticus*[27,41]. The epidemiology of *Yersinia ruckeri*, which started as a geographically isolated disease and quickly became disseminated [31], also was considered to support the epidemic model [27].

Pulsed-field gel electrophoretic analysis of *Y. ruckeri* isolates resulted in a discriminatory index (*D*) of 0.790, whereas repetitive sequence-based PCR approaches yielded low *D* values between 0.048 and 0.140. As the *D* value describes the probability that two randomly selected isolates show different profiles by the method applied, methods with low *D* values are considered not to be sufficiently discriminative for the detection of differences among *Y. ruckeri* isolates. In a previous analysis, “medium to high” *D* values ranging between 0.71–0.74 were obtained by the API 20E, lipopolysaccharide and outer membrane protein analysis [27], while ERIC-and REP-PCR were valuable in combination with other techniques. Similar results were reported from *Y. ruckeri* isolated in Turkey [42]. The combination of several typing methods, as performed by Bastardo *et al*. [27] showed that isolates with similar characteristics were associated with certain fish species and/or predominated in some geographical areas. In the present study, the combination of six typing methods, including API 20E, PFGE and four different repetitive sequence PCR techniques facilitated the identification of 27 different groups. With this set of methods, most of the isolates from rainbow trout (97.3%) could be differentiated from the isolates obtained from pike, koi and brown trout. Biotype 2 isolates were only detected in rainbow trout in this study. The genomic variation of *Y. ruckeri* isolates provides information for comparing their distribution and the relationships among individual isolates from different regions in north west Germany.

## Conclusions

Most of the isolates from north west Germany belonged to biotype 2. The combination of different typing methods could well differentiate *Y. ruckeri* isolates. The results of this study showed the diversity of isolates present in north west Germany. Moreover, these data enable the identification and in-depth characterisation of pathogenic *Y. ruckeri* isolates. As a consequence of this detailed characterisation, these data can be used to examine the distribution of these isolates in the field and may be used as a basis for preventive disease monitoring plans.

## Methods

### Bacterial isolates and sample collection

In total, 83 *Yersina ruckeri* isolates were analysed, including 33 isolates from the strain collection of LAVES Niedersachsen, Food and Veterinary Institute Braunschweig/Hannover, Germany and the fish disease service at the Landesbetrieb Hessisches Landeslabor (LHL) Giessen, Germany. These bacteria were isolated from diseased rainbow trout suffering from ERM outbreaks. In addition, 48 isolates were sampled from rainbow trout (*Oncorhynchus mykiss*) in trout hatcheries at different sites in the German federal state NRW during the four seasons of 2011–2012. Sampling was done under the approval of Landesamt für Natur, Umwelt und Verbraucherschutz, NRW. Fish were randomly sampled when diseased fish were not present in the farm. The sampling was done following the guidelines for the diagnostic of fish diseases and regarded international and national guidelines for animal welfare. Of the remaining two isolates, one was the *Y. ruckeri* reference strain DSM 18,506 and the other a non-motile isolate also from NRW in 2008 and provided by Dr. Gould from MSD Animal Heath. All isolates were kept as glycerol stock cultures at−80°C. All isolates were cultured on trypticase soy agar (TSA; Sigma) at 25°C for 24–48 h and pure cultures were kept at−80°C using Cryobank beads (Copan Diagnostic INC., USA).

### Identification of *Y. ruckeri*

All *Y. ruckeri*-suspect cultures were examined by Gram-staining and colony morphology. For species confirmation, the pure-cultured colonies were biochemically characterised by the API 20E system at 25°C for 48 h (bioMérieux, Craponne, France), as well as subjected to the following standard biochemical tests: cytochrome-oxidase production, catalase production, Methylred/Voges-Proskauer (VP), growth on McConkey agar, xylose utilisation, glucose utilisation/gas production, tween 80/20 hydrolysis, nitrate production, citrate utilisation, sorbitol fermentation, gelatine hydrolysis, and oxidative/fermentative glucose utilisation. Motility was checked by phase-contrast microscopy (1000×). To confirm the motility, bacteria were stab-inoculated with a needle into the bottom of API M medium (bioMérieux). In addition, silver staining [43] was used to investigate the isolates for the presence of flagella. After staining, the slides were microscopically inspected (Leica DFC320, Bensheim, Germany).

### DNA extraction

Bacterial cells were lysed and genomic DNA was extracted, following the manufacturers protocol provided in the innuPREP Bacteria DNA Kit (Analytik Jena, Jena, Germany). The purity of DNA was confirmed using a NanoDrop spectrophotometer (NanoDrop, Delaware, USA).

### Repetitive sequence-based PCR approaches

For genotyping, four different repetitive sequence-based PCRs were performed including BOX-A1R-based repetitive extragenic palindromic-PCR (BOX-PCR), (GTG)_5_-PCR, enterobacterial repetitive intergenic consensus (ERIC-PCR) and repetitive extragenic palindromic (REP-PCR). These PCR assays were performed for all *Y. ruckeri* isolates [44], using primers listed in Table 3. PCRs were performed in 25 μl volumes containing 2 μl of DNA template, 0.2 mM concentrations of deoxynucleoside triphosphates, 2.5 μl of 10 × PCR buffer (Qiagen, Hilden, Germany), 2 mM MgCl_2_, 8 μM, 1 μM and 2 μM of each forward and reverse primer for BOX-/(GTG)_5_-, ERIC-and REP-PCR, respectively, and 5 U of Qiagen HotstarTaq DNA Polymerase (Qiagen). The reaction was performed in a Biometra T3000 thermocycler (Analytik Jena, Germany) with an initial denaturation cycle at 95°C for 7 min, followed by 30 cycles of amplification (denaturation for 1 min at 94°C, annealing for 1 min at 53°C, 52°C and 44°C for BOX, ERIC/(GTG)_5_, and REP-PCR, respectively, and extension for 8 min at 65°C), and a final elongation for 16 min at 65°C. The PCR products were detected by electrophoresis by using 2.5% agarose gels. The images obtained were analysed by the UPGMA method using GelCompar II (Applied Maths, Belgium).

**Table 3:** Primers used in this study

**PCR**	**Name**	**Sequence (5′ → 3′)**	**References**
Specific PCR of *Y. ruckeri*	YER8	GCGAGGAGGAAGGGTTAAGTG	[45]
	YER10	GAAGGCACCAAGGCATCTCTG	[45]
(GTG)_5_-PCR	(GTG)_5_	GTGGTGGTGGTGGTG	[44]
BOX-PCR	BOXA1R	CTACGGCAAGGCGACGCTGACG	[44]
ERIC-PCR	ERIC2	AAGTAAGTGACTGGGGTGAGCG	[44]
	ERIC1R	ATGTAAGCTCCTGGGGATTCAC	[44]
REP-PCR	REP2-I	ICGICTTATCIGGCCTAC	[44]
	REP1R-I	IIIICGICGICATCIGGC	[44]

### 16S rDNA gene sequencing

A specific PCR for the identification of *Y. ruckeri* was applied to five isolates which showed patterns different from most of the isolates in repetitive sequence-based PCR. For this PCR, the primers YER8 and YER10 (listed in Table 3) were used according to Gibello *et al.*[45]. The PCR was performed using the Titanium Taq PCR Kit according to the manufacturer’s protocol (Clontech, Saint-Germain-en-Laye, France). Briefly, the PCR was performed in 20 μl volumes containing 1 μl of DNA template, 0.2 mM deoxynucleoside triphosphates, 2 μl of 10× Titanium Taq buffer (Clontech, USA), 10 μM of each forward and reverse primer, and 0.1 μl of 50 × Titanium Taq DNA Polymerase (Clontech). The reaction was performed in a BIORON thermocycler (BIORON, Germany) with an initial denaturation cycle for 3 min at 95°C, followed by 40 cycles of amplification (denaturation for 30 s at 95°C, annealing for 30 s at 60°C, and extension for 1 min at 72°C), and a final elongation for 7 min at 72°C. PCR products were sent to LGC Genomics (Berlin, Germany) for sequencing. The resulting 16S rDNA sequences were compared in a BLAST search with the sequences previously deposited in the NCBI database (http://www.ncbi.nlm.nih.gov).

### Pulsed-field gel electrophoresis (PFGE)

Isolates to be examined by pulsed-field gel electrophoresis were grown on TSA plates for 48 h at 25°C. Agarose plugs containing *Yersinia ruckeri* genomic DNA were prepared according to the method of Wagley *at el*[39]. Slices of the agarose plugs that contain *Y. ruckeri* genomic DNA were digested with the restriction endonuclease *NotI* (100 U) for 4 h at 37°C. A *Salmonella* Braenderup H9812 molecular standard was prepared by the same method and restricted with *Xba*I [46]. *NotI* digested agarose plugs were loaded into a 1% (w/v) PFGE-grade agarose gel (Roth, Germany) and subjected to electrophoresis in 0.5 × TBE buffer using a Bio-Rad CHEF-DRIII system. PFGE conditions were 5.6 V/cm with switch times of 1 to 15 s at 14°C for 20 h. Images were captured under ultraviolet light. The images obtained were analysed by the UPGMA method using GelCompar II (Applied Maths).

### Discriminatory power of typing methods

Simpson’s index of diversity was applied to calculate the discriminatory power of the different typing methods: API 20E, REP-PCR, BOX-PCR, (GTG)_5_-PCR, ERIC-PCR and PFGE according to Hunter and Gaston [47].

## Competing interests

The authors declare that they have no competing interests.

## Authors’ contributions

YH, MR, SS and DS designed the study. YH carried out the biochemical identification and repetitive sequence-based PCRs according to the suggestions from MR and AJ. GBM and YH performed the PFGE and analysed the results under the supervision of SS and DS. YH drafted the manuscript and all authors critically revised the manuscript. All authors read and approved the final manuscript.

## Supplementary Material

Additional file 1: Table S1 Different origins of *Yersinia ruckeri*. Click here for file

## References

[B1] Noga EJ Fish disease: diagnosis and treatment 2010 2 Iowa: Wiley-Blackwell

[B2] Valtonen ET Rintamaki P Koskivaara M Occurrence and pathogenicity of *Yersinia ruckeri* at fish farms in northern and central Finland J Fish Dis 1992 9 163 171 10.1111/j.1365-2761.1992.tb00650.x

[B3] Oraić D Zrnčić S Šoštarić B Bažulić D Lipej Z Occurrence of enteric redmouth disease in rainbow trout(Oncorhynchus Mykiss) on farms in Croatia Acta Vet Hung 2002 9 3 283 291 10.1556/AVet.50.2002.3.4 12237969

[B4] Lesel R Lesel M Outbreak of enteric redmouth disease in rainbow trout, Salmo gairdneri Richardson, in France J Fish Dis 1983 9 385 387 10.1111/j.1365-2761.1983.tb00091.x

[B5] Fuhrmann H Boehm KH An outbreak of enteric redmouth disease in West Germany J Fish Dis 1983 9 309 311 10.1111/j.1365-2761.1983.tb00080.x

[B6] Meier W Enteric redmouth disease: outbreak in rainbow trout in Switzerland Dis Aquat Organ 1986 9 81 82

[B7] Ross AJ Rucker RR Ewing WH Description of a bacterium associated with redmouth disease of rainbow trout (*Salmo Gairdneri*) Can J Microbiol 1966 9 4 763 770 10.1139/m66-103 6007992

[B8] Tebbit GL Erickson JD Vande Water RB Development and use of *Yersinia ruckeri* bacterins to control enteric redmouth disease International symposium on fish biologies: serodiagnostics and vaccines. vol. 49: Developments in biological standardization 1981 395 401

[B9] Davies RL O-Serotyping of *Yersinia ruckeri* with special emphasis on European isolates Vet Microbiol 1990 9 299 307 10.1016/0378-1135(90)90016-O 1694607

[B10] Davies RL Clonal analysis of *Yersinia ruckeri* based on biotypes, serotypes and outer membrane protein-types J Fish Dis 1991 9 221 228 10.1111/j.1365-2761.1991.tb00591.x

[B11] Romalde JL Margarinos B Barja JL Toranzo AE Antigenic and molecular characterization of Yersinia ruckeri: proposal for a new intraspecies classification Syst Appl Microbiol 1993 9 411 419 10.1016/S0723-2020(11)80274-2

[B12] Arias CR Olivares-Fuster O Hayden K Shoemaker CA Grizzle JM Klesius PH First report of *Yersinia ruckeri* biotype 2 in the USA J Aquat Anim Health 2007 9 35 40 10.1577/H06-011.1 18236630

[B13] Fouz B Zarza C Amaro C First description of non-motile *Yersinia ruckeri* serovar I strains causing disease in rainbow trout, *Oncorhynchus mykiss (Walbaum)*, cultured in Spain J Fish Dis 2006 9 339 346 10.1111/j.1365-2761.2006.00723.x 16768714

[B14] Austin DA Robertson PAW Austin B Recovery of a new biogroup of *Yersinia ruckeri* from diseased rainbow trout (*Oncorhynchus mykiss*, Walbaum) Syst Appl Microbiol 2003 9 127 131 10.1078/072320203322337416 12747420

[B15] Wheeler RW Davies RL Dalsgaard I Garcia J Welch TJ Wagley S Bateman KS Verner-Jeffreys DW *Yersinia ruckeri* biotype 2 isolates from mainland Europe and the UK represent different clonal groups Dis Aquat Organ 2009 9 25 33 19419004 10.3354/dao02039

[B16] Welch TJ Verner-Jeffreys DW Dalsgaard I Wiklund T Evenhuis JP Cabrera JAG Hinshaw JM Drennan JD LaPatra SE Independent emergence of *Yersinia ruckeri* biotype 2 in th United States and Europe Appl Environ Microbiol 2011 9 10 3493 3499 10.1128/AEM.02997-10 21441334 PMC3126439

[B17] Klein BU Kleingeld DW Bohm KH First isolation of a non-motile/tween 80 negative *Yersinia ruckeri* strain in Germany Bull Eur Assoc Fish Pathol 1994 9 5 165 166

[B18] Karatas S Candan A Demircan D Enteric red mouth disease in cultured rainbow trout (Oncorhynchus Mykiss) on the black sea coast of Turkey Isr J Aquacult-Bamid 2004 9 3 226 231

[B19] Wang KY Fan FL Huang XL Geng Y Chen DF Huang JL Occurrence and treatment of a channel catfish*(Ictalurus punctatus(Rafinesque)*) disease, caused by *Yersinia ruckeri* Sci Fish Farming 2009 9 50 51

[B20] Coquet L Cosette P Quillet L Petit F Junter GA Jouenne T Occurrence and phenotypic charaterization of *Yersinia ruckeri* strains with biofilm-forming capacity in a rainbow trout Farm Appl Environ Microbiol 2002 9 2 470 475 10.1128/AEM.68.2.470-475.2002 11823180 PMC126714

[B21] Busch RA Enteric redmouth disease(Hagerman strain) Mar Fish Rev 1978 9 42 51

[B22] Roberts MS A report of an epizootic in hatchery-reared rainbow trout, salmo gairdneri Richardson, at an English trout farm, caused by Yersinia ruckeri J Fish Dis 1983 9 551 552 10.1111/j.1365-2761.1983.tb00111.x

[B23] Noga EJ Enteric redmouth disease (ERM, redmouth, yersiniosis, blood spot, yersinia ruckeri infection) 2000 Ames: Iowa State University Press

[B24] Danley ML Goodwin AE Killian HS Epizootics in farm-raised channel catfish, *Ictalurus punctatus(Rafinesque)*, caused by the enteric redmouth bacterium *Yersinia ruckeri* J Fish Dis 1999 9 451 456 10.1046/j.1365-2761.1999.00196.x

[B25] Stevenson RMW Airdrie DE Serological variation among *Yersinia ruckeri* strains J Fish Dis 1984 9 247 254 10.1111/j.1365-2761.1984.tb00930.x

[B26] Davies RL Frerichs GN Morphological and biochemical differences among isolates of *Yersinia ruckeri* obtained from wide geographical areas J Fish Dis 1989 9 357 365 10.1111/j.1365-2761.1989.tb00324.x

[B27] Bastardo A Ravelo C Romalde JL Multilocus sequence typing reveals high genetic diversity and epidemic population structure for the fish pathogen *Yersinia ruckeri* Environ Microbiol 2012 9 8 1888 1897 10.1111/j.1462-2920.2012.02735.x 22463110

[B28] Schill WB Phelps SR Pyle SW Multilocus electrophoretic assessment of the genetic structure and diversity of *Yersinia ruckeri* Appl Environ Microbiol 1984 9 5 975 979 16346669 10.1128/aem.48.5.975-979.1984 PMC241660

[B29] Lucangeli C Morabito S Caprioli A Achene L Busani L Mazzolini E Fabris A Macri A Molecular fingerprinting of strains of *Yersinia ruckeri* serovar O1 and *Photobacterium damsela* subsp *piscicida* isolated in Italy Vet Microbiol 2000 9 3 273 281 10.1016/S0378-1135(00)00241-8 10973701

[B30] Bastardo A Ravelo C Romalde JL A polyphasic approach to study the intraspecific diversity of *Yersinia ruckeri* strains isolated from recent outbreaks in salmonid culture Vet Microbiol 2012 9 1–2 176 182 22721731 10.1016/j.vetmic.2012.05.024

[B31] Austin B Austin DA Bacterial fish pathogens: diseases of farmed and wild fish 2007 Chichester, UK: Praxis Publishing

[B32] Sousa JA Magarinos B Eiras JC Toranzo AE Romalde JL Molacular characterization of Portuguese strains of *Yersinia ruckeri* isolated from fish culture systems J Fish Dis 2001 9 151 159 10.1046/j.1365-2761.2001.00281.x

[B33] Bastardo A Sierralta V Leon J Ravelo C Romalde JL Phenotypical and genetic characterization of *Yersinia ruckeri* strains isolated from recent outbreaks in farmed rainbow trout *Oncorhynchus mykiss* (Walbaum) in Peru Aquaculture 2011 9 229 232 10.1016/j.aquaculture.2011.03.040

[B34] Bastardo A Bohle H Ravelo C Toranzo AE Romalde JL Serological and molecular heterogenetity among *Yersinia ruckeri* strains isolated from farmed Atlantic salmon *Salmo salar* in Chile Dis Aquat Organ 2011 9 207 214 10.3354/dao02296 21516973

[B35] Wortberg F Nardy E Contzen M Rau J Identification of Yersinia ruckeri from diseased salmonid fish by fourier transform infrared spectroscopy J Fish Dis 2012 9 1 10 10.1111/j.1365-2761.2011.01317.x 22103737

[B36] Furones MD Rodgers CJ Munn CB Yersina ruckeri, the causal agent of enteric redmouth disease(ERM) in fish Annu Rev of Fish Dis 1993 9 105 125

[B37] Aral H Morita Y Izumi S Katagiri T Kimura H Molecular typing by pulsed-field gel electrophoresis of *Flavobacterium psychrophilum* isolates derived from Japanese fish J Fish Dis 2007 9 345 355 10.1111/j.1365-2761.2007.00809.x 17498178

[B38] Soto E Mauel M Lawrence M Improved pulsed-field gel electrophoresis procedure for the analysis of *Flavobacterium columnare* isolates previously affected by DNA degration Vet Microbiol 2008 9 207 212 10.1016/j.vetmic.2007.10.001 18023300

[B39] Wagley S Koofhethile K Wing JB Rangdale R Comparison of V parahaemolyticus isolated from seafoods and cases of gastrointestinal disease in the UK Int J Environ Health Res 2008 9 4 283 293 10.1080/09603120801911064 18668416

[B40] Smith JM Smith MH O’Rourke M Spratt BG How clonal are bacteria? Proc Natl Acad Sci U S A 1993 9 10 4384 4388 10.1073/pnas.90.10.4384 8506277 PMC46515

[B41] Gonzalez-Escalona N Martinez-Urtaza J Romero J Espejo RT Jaykus LA DePaola A Determination of molecular phylogenetics of Vibrio parahaemolyticus strains by multilocus sequence typing J Bacteriol 2008 9 8 2831 2840 10.1128/JB.01808-07 18281404 PMC2293261

[B42] Onuk EE Ciftci A Findik A Ciftci G Altun S Balta F Ozer S Coban AY Phenotypic and molecular characterization of *Yersinia ruckeri* isolates from rainbow trout (*Oncorhynchus mykiss*, Walbaum,1792) in Turkey Berl Munch Tierarztl Wochenschr 2011 9 320 328 21848040

[B43] Blenden DC Goldberg HS Silver impregnation stain for *Leptospira* and flagella Am Soc Microbiol 1965 9 3 899 900 10.1128/jb.89.3.899-900.1965 PMC277553 14273677

[B44] Amann S Untersuchung zur Klassifizierung, identifizierung und differenzierung von Yersinia Arten 2007 Wien: University Wien

[B45] Gibello A Blanco MM Moreno MA Cutuli MT Domenech A Moniguez L Fernandez-Garayzabal JF Development of a PCR assay for detection of *Yersinia ruckeri* in tissues of inoculated and naturally infected trout Appl EnvironMicrobiol 1999 9 1 346 350 10.1128/aem.65.1.346-350.1999 PMC91030 9872807

[B46] Hunter SB Vauterin P Lambert-Fair MA VanDuyne MS Establishment of a universal size standard strain for use with the PulseNet standardized pulsed-field get electrophoresis protocols: converting the national databases to the new size standard J Clin Microbiol 2005 9 1045 1050 10.1128/JCM.43.3.1045-1050.2005 15750058 PMC1081233

[B47] Hunter PR Gaston MA Numerical index of the discriminatory ability of typing systems: an application of simpson’s index of diversity J Clinical Microbiol 1988 9 11 2465 2466 3069867 10.1128/jcm.26.11.2465-2466.1988 PMC266921

