# Supplementary material for: Biochemical and molecular heterogeneity among isolates of Yersinia ruckeri from rainbow trout (Oncorhynchus mykiss, Walbaum) in north west Germany
Source: BMC Vet Res. 2013 Oct 21;9:215. doi: 10.1186/1746-6148-9-215 (PMC4016151; doi:10.1186/1746-6148-9-215)
Supplement: Additional file 1: Table S1 — Different origins of Yersinia ruckeri. [file 1746-6148-9-215-S1.doc]

Table S1: Different origins of *Yersinia ruckeri*

| **Typing Group (tp)** | **No. of isolates** | **Origins** | **Isolated year** |
| --- | --- | --- | --- |
| tp1 | 5 | **LS** | 2005  2006  2009 |
|  |  | **H** | 2010 |
|  |  | B | 08/2011 |
| tp2 | 21 | **LS** | 2005  2007 |
|  |  | **H** | 2010 |
|  |  | SR | 06/2011  09/2011  06/2012 |
|  |  | KP | 08/2011 |
|  |  | P | 09/2011  06/2011 |
|  |  | A | 09/2011 |
| tp3 | 1 | SR | 08/2011 |
| tp4 | 1 | B | 08/2011 |
| tp5 | 2 | **LS** | 2009 |
|  |  | B | 09/2011 |
| tp6 | 8 | **LS** | 2005  2007 |
|  |  | **H** | 2010 |
|  |  | P | 06/2011 |
|  |  | SR | 02/2012 |
| tp7 | 1 | KP | 02/2012 |
| tp8 | 7 | **NRW** | 2008 |
|  |  | KP | 02/2012 |
|  |  | SR | 04/2012  06/2012 |
|  |  | ST | 04/2012 |
| tp9 | 1 | **LS** | 2004 |
| tp10 | 1 | **LS** | 2007 |
| tp11 | 1 | **H** | 2010 |
| tp12 | 1 | **LS** | 2008 |
| tp13 | 2 | **LS** | 2005  2007 |
| tp14 | 1 | **LS** | 2005 |
| tp15 | 1 | P | 06/2011 |
| tp16 | 3 | L | 06/2011  08/2011 |
| tp17 | 1 | B | 06/2011 |
| tp18 | 7 | **LS** | 2004  2005  2007 |
|  |  | SR | 06/2011 |
| tp19 | 2 | **LS** | 2004  2007 |
| tp20 | 7 | SR | 06/2011  08/2011 |
|  |  | F | 06/2012 |
|  |  | B | 09/2011  02/2012  04/2012 |
|  |  | A | 09/2011 |
| tp21 | 2 | N | 09/2011 |
| tp22 | 1 | P | 09/2011 |
| tp23 | 1 | **LS** | 2005 |
| tp24 | 1 | **H** | 2011 |
| tp25 | 1 | **LS** | 2005 |
| tp26 | 2 | **LS** | 2005 |
|  |  | KP | 06/2012 |
| tp27 | 1 (DSM18506)*^b^* | - | - |

**LS**: Lower Saxony; **H**: Hessen; **NRW**: North Rheine-Westphalia (non-motile isolate from 2008)

SR, KP, P, L, ST, A, B, N, F are the fish farms located in North Rheine-Westphalia where the samples were collected during 2011-2012.
